# Neglected huge chest wall mass. A case report of fibrous dysplasia

**DOI:** 10.1016/j.ijscr.2023.107912

**Published:** 2023-02-03

**Authors:** Ershadi Reza, Amini Hesam, Soltanmohammadi Sara, Rafieian Shahab

**Affiliations:** a Department of Thoracic Surgery, Imam Khomeini Hospital Complex, Tehran University of Medical Sciences, Tehran, Iran; b Department of Pulmonology, Imam Khomeini Hospital Complex, Tehran University of Medical Sciences, Tehran, Iran

**Keywords:** Fibrous dysplasia, Chest wall tumor, Thoracotomy, Case report

## Abstract

**Introduction:**

fibrous dysplasia is a slow-growing bone tumor and is caused by the failure of bone maturation. It is usually asymptomatic thus it is generally found incidentally in radiologic evaluations. Computed tomography is the best radiologic modality for its evaluation. The characteristic findings are ground-glass lesions surrounded by a rim or shell of reactive bone.

**Presentation of case:**

This study presents a 52-year-old male patient with a huge chest wall tumor arising from the posterolateral aspect of the right fourth to seventh ribs measuring 38 cm. He underwent a thoracotomy and the tumor was resected. For safe margin, the third and eighth ribs were also resected. The defect was reconstructed with a prolene mesh patch and a pectoralis major flap. The final pathology report stated a tumor composed of spindle cells without pleomorphism or mitotic figures with intervening branching and anastomosing bone trabeculae. The margins were tumor-free and on the follow-up, the patient's condition was decent.

**Discussion:**

Primary tumors of the rib account for 5 % to 7 % of all primary bone neoplasms. Fibrous dysplasia makes up 0.8 % of primary bone tumors. Fibrous dysplasia usually causes no symptoms although it can get massive enough to get symptomatic. Its diagnosis is made through clinical, radiological, and histopathological investigations. CT scan findings are the cornerstone for radiologic evaluations. An individualized approach based on the patient's age and symptoms should be considered.

**Conclusion:**

Considering that the malignant degeneration of the tumor is uncommon, early diagnosis and surgical resection of the tumor can be curative.

## Highlights

• A 52-year-old male patient with a huge chest wall tumor arising from the right fourth to seventh ribs
• Histology: spindle cells without pleomorphism or mitotic figures with intervening branching and anastomosing bone trabeculae
• An intramedullary lesion with a ground-glass center and cortical thickening measuring 38 cm in diameter
• Primary tumors of the rib constitute 5% to 7% of all primary bone neoplasms.
• Fibrous dysplasia usually produces no symptoms. The lesions are detected accidentally during radiological investigations.

## 1 Introduction

Fibrous dysplasia first came to notice in 1938 by Lichtenstein [1]. It occurs as a result of bone maturation failure from the primitive stage to the lamellar stage. Bone mineralization and remodeling are also impaired under mechanical stress [2]. Although its etiology is not entirely recognized, mutation of signal-transducing G proteins encoded by GNAS1 on chromosome 20 is likely associated with fibrous dysplasia. Malignant degeneration is uncommon [3]. The disease affects men and women equally and can occur at any age although the peak prevalence is in the third decade of life [4]. Based on systemic findings and bone involvement, fibrous dysplasia is categorized into four distinct classes: single bone involvement (monostotic), multiple bone involvement (polyostotic), or accompanying McCune-Albright or Mazabraud disease [5], [6], [7]. Monostotic fibrous dysplasia is the most common form of the disease which is usually asymptomatic and is found incidentally. However, based on the size and location of the tumor, it can present itself with varying symptoms such as bone pain, deformity, stress fractures, and pathologic fractures [5], [8], [9], [10].

Radiologic studies can facilitate the diagnosis. Bone lesions have ground-glass patterns in plain x-ray images due to the replacement of normal bone with radiolucent tissue. Bone lesions are surrounded by rims or shells of reactive bone and are a characteristic finding [5], [11]. Bone scintigraphy can help estimate the severity of the disease. The lesions have high radionucleotide uptake but it diminishes over time as the lesions mature [12]. Computed tomography is the best modality for evaluation since the lesions enhance in a contrast study [5]. Magnetic resonance imaging can add some useful information to CT scan findings [13]. We present a case of fibrous dysplasia with a huge chest wall mass causing dyspnea. This work has been reported in line with the SCARE criteria [14].

## 2 Case presentation

**Fig. 1:**
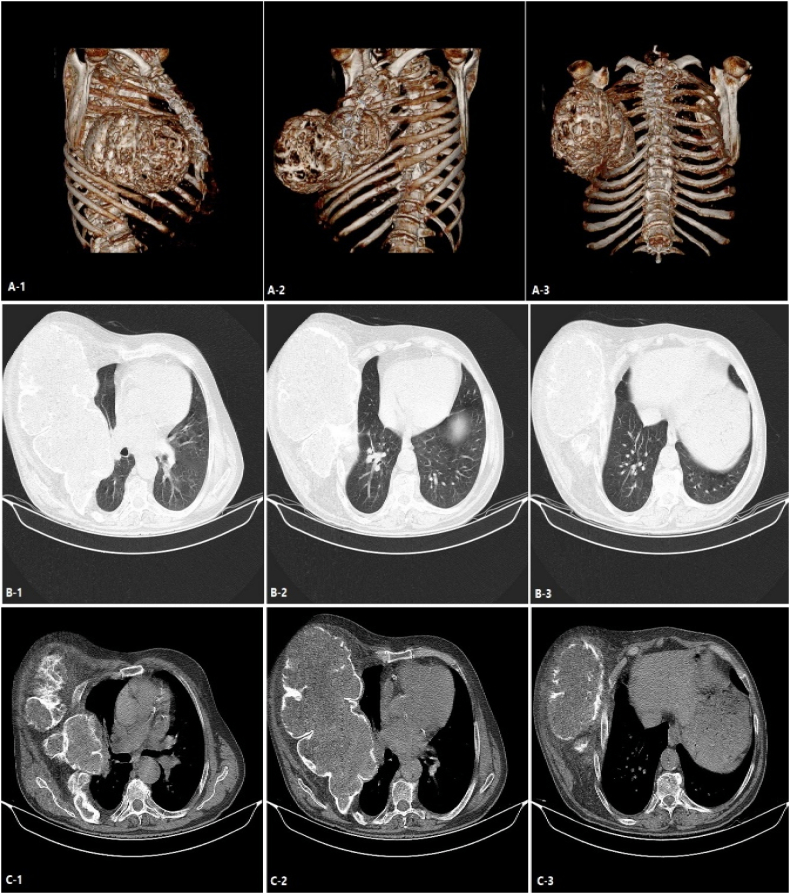
Computed tomography scan demonstrating a 36 × 18 cm mass with a rim of reactive bone. *A*1–3: Reconstructed Images. *B*1–3: Parenchymal window images *C*1–3: Mediastinal window images.

**Fig. 2:**
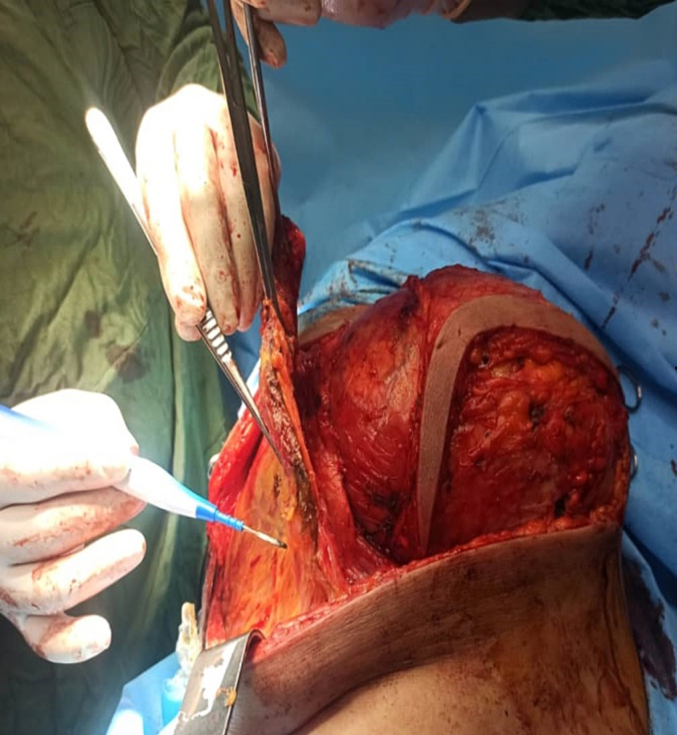
Resection of the tumor.

**Fig. 3:**
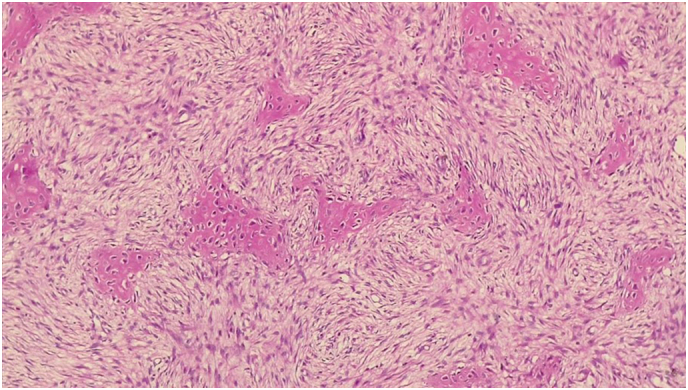
Photomicrograph showing spindle cells without pleomorphism or mitotic figures with intervening branching and anastomosing bone trabeculae (H & E).

**Fig. 4:**
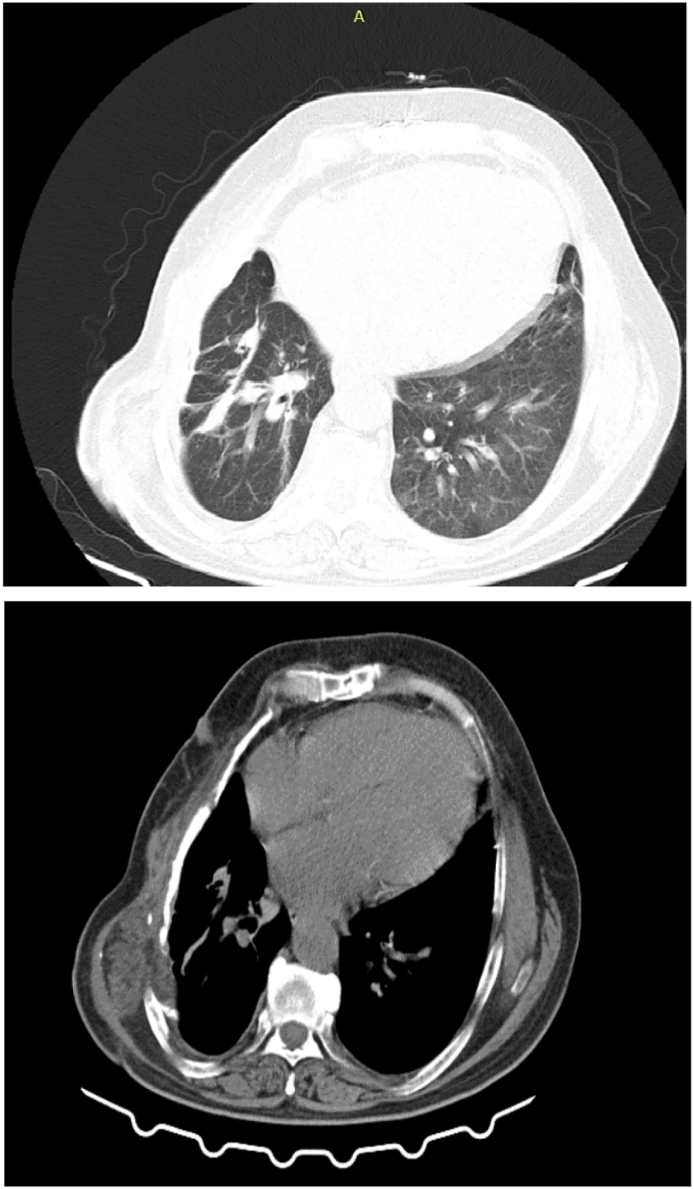
Post-operative CT scan.

This study presents a 52-year-old middle-eastern male patient with a huge slow-growing chest wall mass located on the right chest wall. His past medical and surgical history was unremarkable and he had no known allergies albeit he was a smoker. The patient's vital signs were all within the normal range (blood pressure 110/75 mmHg, pulse rate 76 bpm, respiratory rate 17 rpm, temperature 37.3 °C, blood oxygen saturation 95 %). Except for the large firm chest wall mass located on the right chest wall, his physical examination was unremarkable. Chest X-ray and computed tomography (CT) displayed a giant intramedullary lesion with a ground-glass center and thickening of the cortex arising from the posterolateral aspect of the right fourth to seventh ribs measuring 38 cm in diameter. The pulmonary function test (PFT) of the patient, who was a smoker, revealed the following: FEV1, 55 %; FVC, 53 %; FEV1/FVC 0.79; FEF25–75: 47 %. A 3-dimensional reconstruction of the CT scan was performed and in Fig. 1 the size and the extension of the lesion can be seen. A solid lesion, which destroyed the fourth to seventh ribs with axial dimensions of approximately 36 × 18 cm was detected in the CT scan. The previous pathology report was inconclusive and reported a spindle cell lesion. The patient was discussed with a multidisciplinary tumor board including thoracic surgeons and oncologists then they agreed upon a surgical excision to diagnose and treat the patient. Anesthesia was induced and the patient was intubated with a double-lumen endotracheal tube. The patient was put in the left lateral decubitus position. Thoracotomy was initiated by an elliptical incision. The thorax was opened with a right lateral thoracotomy. Thereafter, we observed a mass measuring about 36 × 18 × 11 cm in dimensions adhering to the fourth to seventh ribs. It had adhesion neither to the lung nor the heart. The tumor was resected and for safe margins, the third and eighth ribs were also resected (Fig. 2). The defect size was 40 × 25 cm in diameter. The defect was reconstructed with a prolene mesh patch and bone cement. The mesh was then covered with pectoralis major pedicled flap. A thoracic tube was placed in the apex of the lung. The patient was monitored in the ICU for two days and then was transferred to the thoracic surgery ward on the third day. The thoracic tube, discharging no fluids, was removed on the fourth day. Finally, the patient was discharged from the hospital on the sixth day. The patient was followed up periodically. The final pathology report stated: “a tumor composed of spindle cells without pleomorphism or mitotic figures with intervening branching and anastomosing bone trabeculae”. Bone trabeculae were woven without a prominent osteoblastic rim. Furthermore, the margins were tumor-free (Fig. 3). On the follow-up, after 30 days, the patient's condition was excellent. He had no complaints and his wounds had healed. The post operation CT scan release expanded lung and normal chest wall (Fig. 4).

## 3 Discussion

Primary chest wall tumors are infrequent. Primary tumors of the rib constitute 5 % to 7 % of all primary bone neoplasms. Benign bone tumors of the chest wall consist of osteochondroma, fibrous dysplasia, chondroma, and eosinophilic granuloma [15].

Fibrous dysplasia accounts for 0.8 % of primary bone tumors and was first described by Lichtenstein in 1938. It is characterized by the replacement of normal bone with fibrous tissue and immature bone structures [1]. The likely etiology is mutations in GNAS gene 20q. This gene encodes Gs alpha in the target cells which are responsible for bone cell alternations [3].

Fibrous dysplasia usually produces no symptoms. The lesions are detected accidentally during radiological investigations. However, in rare cases, they may become symptomatic as they grow in size. They can also cause pathologic fractures, neuropathy, and deformities [16].

Clinical, radiologic, and histopathological investigations are the pillars for the diagnosis of fibrous dysplasia. CT scan findings are the cornerstone of radiologic evaluations. The “ground-glass” appearance is the result of medullary space replacement by the mixture of woven bone and fibrous components [3]. Polymerase chain reaction (PCR) can be utilized for the diagnosis. Missense point mutations in the G_s_α at the Arg^201^ codon are positive and the tumor cells do not express the proliferating cell nuclear antigen [17]. Histologic features consist of cellular fibrous dysplasia containing a proliferation of bland and uniform spindle cells with sparse mitotic activity [18].

In an asymptomatic patient with typical radiologic features consistent with the diagnosis of fibrous dysplasia, a biopsy can be withheld. In that case, conservative management with a close clinical and radiological assessment for tumor progression is reasonable [5]. Newly diagnosed patients with multiple lesions should be evaluated to rule out metabolic and endocrine disorders accompanying the disease [5]. Bisphosphonates have shown promising results in the medical treatment of the symptomatic polyostotic variant of the disease [19].

Surgical intervention is indicated when symptomatic lesions or deformities and pathologic fractures are present. Regarding skeletal maturity and the possibility of tumor progression and maturation, age is an important factor in decision-making for surgical treatment [11], [12].

Complete resection of the lesion is sufficient and the defect has to be reconstructed. In our case, we used both a pectoralis major flap and a prolene mesh. As far as we know, few reports have described huge fibrous dysplasia of the chest wall. Mahadevappa et al. reported a case report of a 70-year-old female with a chest wall mass measuring 10 × 9 cm in size, However, this tumor was smaller than the one we observed in our case [20].

## 4 Conclusion

Fibrous Dysplasia is a rare bone tumor with slow progression. Considering that the malignant degeneration of the tumor is uncommon, early diagnosis and surgical resection of the tumor can be curative in symptomatic patients.

## Consent

Written informed consent was obtained from the patient for publication of this case report and accompanying images. A copy of the written consent is available for review by the Editor-in-Chief of this journal on request.

## Ethical approval

Investigations were in accordance with Helsinki Declaration of 1964 and all subsequent revisions.

## Sources of funding

This study did not receive any specific backing from funding agencies in the public, commercial, or non-profit sectors.

## Guarantor

Amini Hesam.

## Research registration

N/A.

## CRediT authorship contribution statement

**Ershadi Reza:** Writing – original draft, Resources. **Amini Hesam:** Conceptualization, Writing – original draft, Investigation, Resources, Methodology, Validation, Supervision, Writing – review & editing. **Soltanmohammadi Sara:** Writing – original draft, Resources. **Rafieian Shahab:** Writing – original draft, Resources.

## Declaration of competing interest

N/A.

## References

[1] Lichtenstein L. (1942). Fibrous dysplasia of bone. Arch. Pathol..

[2] Enneking W.F., Rathe R., Cornwall G. (1998).

[3] Singer F.R. (1997 Dec). Fibrous dysplasia of bone: the bone lesion unmasked. Am. J. Pathol. [Internet].

[4] DiCaprio M.R. (2005). Fibrous dysplasiapathophysiology, evaluation, and treat<sbt aid="1027210">ment </sbt>. J. Bone Joint Surg..

[5] Henry A. (1969 May). Monostotic fibrous dysplasia. J. Bone Joint Surg. Br..

[6] Wirth W.A., Leavitt D., Enzinger F.M. (1971 May). Multiple intramuscular myxomas. Another extraskeletal manifestation of fibrous dysplasia. Cancer [Internet]..

[7] Blasier R.D., Ryan J.R., Schaldenbrand M.F. (1986). Multiple myxomata of soft tissue associated with polyostotic fibrous dysplasia. A case report. Clin Orthop Relat Res [Internet]..

[8] Ippolito E., Bray E.W., Corsi A., de Maio F., Exner U.G., Robey P.G. (2003 May). Natural history and treatment of fibrous dysplasia of bone: a multicenter clinicopathologic study promoted by the European Pediatric Orthopaedic Society. J. Pediatr. Orthop. B [Internet].

[9] Nakashima Y., Kotoura Y., Nagashima T., Yamamuro T., Hamashima Y. (1984). Monostotic fibrous dysplasia in the femoral neck. A clinicopathologic study. Clin Orthop Relat Res [Internet]..

[10] Kaplan F.S., Fallon M.D., Boden S.D., Schmidt R., Senior M., Haddad J.G. (1988 Aug 18). Estrogen receptors in bone in a patient with polyostotic fibrous dysplasia (McCune-Albright syndrome). N. Engl. J. Med. [Internet].

[11] Fitzpatrick K.A., Taljanovic M.S., Speer D.P., Graham A.R., Jacobson J.A., Barnes G.R. (2004 Jun). Imaging findings of fibrous dysplasia with histopathologic and intraoperative correlation. AJR Am. J. Roentgenol. [Internet].

[12] Zhibin Y., Quanyong L., Libo C., Jun Z., Hankui L., Jifang Z. (2004 Mar). The role of radionuclide bone scintigraphy in fibrous dysplasia of bone. Clin. Nucl. Med. [Internet].

[13] Jee W.H., Choi K.H., Choe B.Y., Park J.M., Shinn K.S. (1996 Dec). Fibrous dysplasia: MR imaging characteristics with radiopathologic correlation. AJR Am. J. Roentgenol. [Internet].

[14] Agha R.A., Franchi T., Sohrabi C., Mathew G., Kerwan A., Thoma A. (2020 Dec). The SCARE 2020 guideline: updating consensus Surgical CAse REport (SCARE) guidelines. Int. J. Surg..

[15] Chen Y.R., Chang C.N., Tan Y.C. (2006). Craniofacial fibrous dysplasia: an update. Chang Gung Med. J..

[16] Favus M.D. (2005). Harrison’s principles of internal medicine.

[17] Maki M., Saitoh K., Horiuchi H., Morohoshi T., Fukayama M., Machinami R. (2001 Aug). Comparative study of fibrous dysplasia and osteofibrous dysplasia: histopathological, immunohistochemical, argyrophilic nucleolar organizer region and DNA ploidy analysis. Pathol. Int..

[18] Aydın O., Barış S., Kefeli M., Şenel A., Yıldız L., Kandemir B. (2009). Fibröz Displazi(Olgu Bildirimi). Journal of Experimental and Clinical Medicine.

[19] Zacharin M., O’Sullivan M. (2000 Sep). Intravenous pamidronate treatment of polyostotic fibrous dysplasia associated with the McCune Albright syndrome. J. Pediatr. [Internet].

[20] Mahadevappa A., Patel S., Ravishankar S., Manjunath G.V. (2012). Monostotic fibrous dysplasia of the rib: a case report. Case Rep Orthop [Internet]..

